# EGFR Expression and KRAS and BRAF Mutational Status in Intestinal-Type Sinonasal Adenocarcinoma

**DOI:** 10.3390/ijms14035170

**Published:** 2013-03-04

**Authors:** Vanessa Szablewski, Jérôme Solassol, Flora Poizat, Marion Larrieux, Louis Crampette, Alain Mange, Caroline Bascoul-Mollevi, Valérie Costes

**Affiliations:** 1 Department of Biopathology, Centre Hospitalier Universitaire de Montpellier, Montpellier 34000, France; E-Mails: j-solassol@chu-montpellier.fr (J.S.); f-poizat@chu-montpellier.fr (F.P.); m-larieux@chu-montpellier.fr (M.L.); v-costes_martineau@chu-montpellier.fr (V.C.); 2 Faculté de Médecine de Montpellier, Université de Montpellier I, Montpellier 34000, France; E-Mail: alain.mange@uni-montp1.fr; 3 Department of Surgery, Centre Hospitalier Universitaire de Montpellier, Montpellier 34000, France; E-Mail: l-crampette@chu-montpellier.fr; 4 Department of Biostatistic, CRLC Val d’Aurelle, Montpellier 34000, France; E-Mail: caroline.mollevi@montpellier.unicancer.fr

**Keywords:** ITAC, KRAS, BRAF, EGFR expression, prognosis marker

## Abstract

Accumulation of molecular alterations, including EGFR overexpression and mutations in KRAS and BRAF, contribute to colorectal carcinogenesis. Since intestinal-type adenocarcinoma (ITAC) of the nasal cavity and paranasal sinus has morphologic and phenotypic features that are usually indistinguishable from colorectal cancer (CRC), it is likely that both tumor types share equivalent genetic alterations. Data from a series of 43 patients treated surgically for ITAC in Montpellier, France between November 1998 and December 2012 were collected. Tumors were characterized for mutations in *KRAS* and *BRAF* as well as EGFR overexpression. Kaplan-Meier survival curves were constructed using overall survival as the primary end points. Patient survival was analyzed using the hazards ratio. Twenty seven tumors (63%) showed EGFR positivity and 30% exhibited a high expression level (+2/+3). *KRAS* mutations were detected in 43% of cases. *BRAF* mutations were identified in 3.6% of specimens. Patients with age superior to 60 years, metastatic status, and *KRAS* mutations had significant overall survival values (*p* = 0.026, *p* = 0.001 and *p* = 0.03, respectively). Our results indicate that *KRAS* mutations and EGFR expression are frequent in ITAC and that *KRAS* mutations predict good patient prognosis in ITAC. Finally, EGFR directed molecular treatments could be investigated in a subset of patients affected by ITAC.

## 1. Introduction

Intestinal-type adenocarcinoma (ITAC) of the nasal cavity and paranasal sinuses is a rare adenocarcinoma subtype that is closely related to professional exposure to wood or leather dusts [1]. They account for 3% of neoplasms occurring in head and neck regions and represent approximately 10%–20% of neoplasms in the sinonasal tract [2]. Interestingly, these tumors have microscopic features that are usually indistinguishable from colorectal cancer (CRC) and an intestinal-type immunohistochemical profile (CK20+/CK7−/CDX2+/villin+) [3]. Therefore, since numerous studies have clearly demonstrated that monoclonal anti-EGFR antibodies have significant clinical activity when administered in combination with irinotecan as a first- or second-line agent [4], this therapeutic drug has been assessed in the treatment of locally advanced head and neck squamous cell carcinoma (HNSCC) in combination with radiotherapy. *KRAS* and *BRAF* mutations are clearly predictive of the response to anti-EGFR target therapy in CRC. However, these genotypes have been poorly investigated in ITAC, thus hampering potential application of anti-EGFR therapy in intestinal-type adenocarcinoma cases. In this study, our aim was to gain more detailed insight into the etiologic and molecular pathogenesis of ITAC through the analysis of *KRAS* and *BRAF* genotypes in a retrospective cohort (1986–2012) of 43 ITAC. Moreover, we assessed the EGFR and microsatellite instability (MSI) status using an immunohistochemical approach. Considering the aggressiveness of sinonasal ITAC, which is associated with high morbidity mostly due to local recurrence, we then evaluated a possible correlation between these markers and clinicopathological parameters in order to find prognostic factors.

## 2. Results and Discussion

Tissues from sinonasal adenocarcinoma patients were collected between 1986 and 2009 at the Centre Hospitalier Universitaire de Montpellier, France. The cohort consisted of 43 subjects. Three tumors were papillary-type, 25 were colonic-type, 5 were solid-type, and 10 were mucinous-type. The mean age was 66 years (range, 39–80 years). Twenty-six patients had exposure to wood dust. Sex distribution, tumor stage and histopathological type are detailed in Table 1. Six patients presented metastases at the time of diagnosis. Thirty-eight patients received radiotherapy after surgery and one before. Twelve patients received chemotherapy after surgery, and one patient before. Of 43 patients, 24 developed local recurrences or distant metastasis, whereas 17 remained disease free. Sixteen patients died because of cancer complications (local recurrence and intracranial invasion) and nine because of undercurrent causes. The overall median survival in our population was 7.4 years, the overall 5-year survival rate was 62%, and the 1-year and 5-year disease-free survival rates were 22% and 6%, respectively.

First, we assessed the EGFR expression level in ITAC. Overall, 27 tumors showed EGFR positivity (63%), with 14 cases scored as 1+, 9 as 2+, and 4 as 3+. When stratifying the frequency of expression into two groups, negative to low (0 and +1 immunopositivity) and high (+2 and +3 immunopositivity), 30% of the tumors exhibited high EGFR expression (Table 2). Analysis of both hMLH1 and hMSH2 mismatch repair gene expression was successfully performed in 42 of 43 ITAC. All cases showed nuclear immunoreactivity for the 2 proteins in both tumoral tissue and internal control (Table 2). The genomic yield of DNA obtained from the tissue samples was 760.2 ± 423.1 μg/mL. We then determined the *KRAS* and *BRAF* genotypes of our series of 43 ITAC samples using HRM analysis. PCR inhibition was observed in 15 bouin-fixed paraffin-embedded tissues, which were thus excluded from the statistical analysis. For 12 (42.9%) and one (3.6%) specimens of the series, a distinct *KRAS* curve and *BRAF* curve patterns were noted on normalized difference plots, and the corresponding curve patterns for the HRM difference plots clearly revealed the HRM-positive samples. The status of *BRAF* and *KRAS* observed by HRM was confirmed by allele-specific PCR in all cases (Table 2).

**Figure 1:**
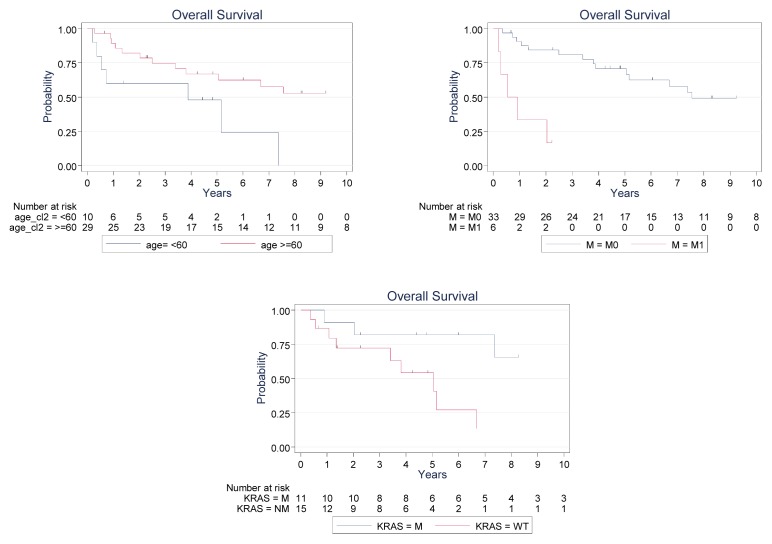
Kaplan–Meier plots of survival according to age, metastasis, and *KRAS* status.

Lastly, we assessed EGFR expression, *KRAS* and *BRAF* mutations and other clinico-histological features relative to overall survival. As expected, age at diagnosis (using <60 years, ≥60 years, HR = 0.35, 95% confidence interval 0.13–0.91, *p* = 0.033), and presence of metastasis at the time of diagnosis (HR = 9.55, 95% confidence interval 2.7–33.4, *p* = 0.0001) were linked with overall survival (Figure 1). Interestingly, the overall 5-year survival rate was 81.8% in *KRAS* wild-type cases *vs.* 54.2% in *KRAS* mutated cases (HR = 3.55, 95% confidence interval 1.06–11.7, *p* = 0.03). Other clinical parameters like wood exposure, tumor stage, histotype, EGFR expression or affected lymph nodes at diagnosis did not show any prognostic value. In addition, when the mucinous histotype was compared to others, univariate analysis did not reveal a significant correlation with overall survival.

According to the WHO histological classification, two main categories of sinonasal adenocarcinoma are recognized: intestinal-type and non-intestinal-type adenocarcinoma. ITAC is a rare neoplasm representing 8%–25% of all malignant sinonasal tumors and it is strongly associated with hard wood dust exposure [5,6]. They morphologically and phenotypically combine both usual types and subtypes of colorectal cancer. There are five sinonasal ITAC pathotype: papillary, colonic, solid, mucinous and mixed [7]. The most frequent type is colonic (40%), followed by solid (20%), papillary (18%) and mucinous and mixed type, representing together 22% of cases. Little is known about genetic changes in ITAC. Because of its histopathological resemblance, most studies so far have focused on genes and proteins often selected for their frequent involvement in colorectal adenocarcinoma. By immunochemical analysis, we found that 63% of ITAC showed EGFR expression and 30% demonstrated high receptor expression. These values are higher than those reported by Franchi *et al.* who found, in their series of 55 ITAC, high expression in 14.5% of the sample [8]. However, they are somewhat similar to those documented in colorectal cancer, with, according to different series, receptor expression is found in approximately 40%–80% of cases [9–11], indicating that both of these tumors have a similar molecular phenotype. However, in previous immunohistochemiscal studies, there was considerable discrepancy in the frequencies of EGFR expression in colorectal cancer, and some found a lower expression rate, sometimes as low as 8% [12]. These discrepancies are probably due to differences in tissue-fixation methods, antibody used, detection techniques and criteria for evaluating the results, which may explain differences between the finding of Franchi *et al.* and ours. In our study, no significant correlation was found with EGFR expression and survival so as A. Franchi *et al.* Nevertheless, the high frequency of EGFR overexpression in ITAC suggests that it has an important role in carcinogenesis of these tumors, but the lack of a consistent correlation with the clinicopathologic features and survival indicate that it is less important in disease progression.

Concordantly to other reports, all hMLH1 and hMSH2 immunophenotype were negative in our cohort. This suggests that unlike colorectal adenocarcinomas, mutations or promoters methylation of DNA mismatch repairs genes and microsatellite instability do not play a role in the pathogenesis of these tumors.

Regarding the *KRAS*/*BRAF* pathway, we investigated the *KRAS* oncogene mutation in codons 12 and 13, which occur in about 40% of colorectal cancers [13–15]. In keeping with the colorectal cancer *KRAS* mutation rate, we found mutations in 42.9% of the tested ITACs. These results are in accordance with Frattini *et al.* and Yom *et al.* who respectively found, 50% (9/18 cases) and 29% (2/7 cases) of *KRAS* mutations in their series of ITAC [16,17], but they contrast with other finding of null or marginal occurrence of *KRAS* in ITAC [5,18–21]. A number of reasons could explain the discrepancy between these results. First, these inconsistencies might be related to different methodological approaches, but not differences in the detection methods used. Effectively, the sensitivity of the combined assays used in all studies should be sufficient to identify mutations in sinonasal tumors. Secondly, they may be merely due to the limited number of patients analysed. Thirdly, the differences in the prevalence of *KRAS* mutations might be related to differences in the extent of wood exposure in the different cohorts. Lopez *et al.* showed that overall or disease-free survival did not differ between *KRAS* mutated and wild-type cases [18]. On the contrary, Pérez *et al.*, who studied the presence of mutations in the 3 ras oncogens (*KRAS*, *NRAS, HRAS*) in 31 cases of ethmoid sinus adenocarcinoma, found *HRAS* mutations in 5/31 cases (16%), and the *HRAS* mutations were related to a worse prognosis. In colorectal cancers, unlike *KRAS*, *BRAF* mutations are associated with clinocopathological features and play a negative prognostic role. Therefore, we focused our analysis on exon 15 of the *BRAF* gene, where the classical V600 mutation is located. We found mutations in one of the ITACs tested. This finding is in agreement with those reported by Lopez *et al.* who reported, in a series of 58 patients, no *BRAF* mutations and confirmed the likely low incidence of *BRAF* mutations in ITAC [18].

Finally, we found a significant correlation with *KRAS* mutations and overall survival (log rank 4.76, *p* = 0.029), as well as for metastatic status, and age. This is the first time that *KRAS* was reported as a prognostic marker in ITAC. *KRAS* was described as an early event in the pathogenesis of colorectal tumors and *KRAS* mutations have been speculated as having a worse prognosis. Unfortunately, the reports have been contradictory on the prognostic value of *KRAS* mutations [22–25]. There have been discrepancies in these reports because of inconsistencies in defining prognosis. Inoue *et al.* reported that *KRAS* mutation was an independent factor associated with prognosis in a multivariate analysis [26], whereas the Kirsten Ras In-Colorectal-Cancer Collaborative Grou*p* (RASCAL) documented the presence of *KRAS* mutation with poorer prognosis [27].

## 3. Experimental Section

### 3.1. Study Population

The study included 43 cases with primary ITAC of the nasal cavity and paranasal sinus, treated in the CENTRE Hospitalier Universitaire de Montpellier between 1986 and 2012. Thirty-eight patients underwent radical surgery, 4 underwent biopsy, and data was unavailable for one patient. Tumor samples were obtained from paraffin-embedded specimens for both immunohistochemical and mutation analyses. Histologic classification was performed according to the World Health Organization guidelines.

Histological slides were reviewed by two pathologists blinded to the clinical data. Patient information was obtained from medical records. Records were reviewed for age, sex, race, history of exposure to dust or smoking, site and stage of tumor, type of treatment, and follow-up status.

### 3.2. EGFR and hMLH1 and hMSH2 Expression Determination

Immunohistochemical analysis was performed on 3 μm sections obtained from the most representative paraffin-embedded tissue block selected on the basis of hematoxylin-eosin stained sections. Immunoperoxydase phenotyping was performed in all cases. For EGFR, analysis was performed using the Dako autostainer and the Anti-EGFR mouse monoclonal antibody Zymed clone 31G7 (1:50 diluted), according to the manufacturer’s instructions. Immunostaining was scored as follows: 0 negative, +1 weak reactivity that was membranous, cytoplasmic or both, +2 circumferential membrane staining with intermediate intensity and frequent cytoplasmic reactivity that was of weaker intensity than the membrane reactivity; and +3 complete strong circumferential staining, usually associated with cytoplasmic staining of weaker intensity.

**Figure 2:**
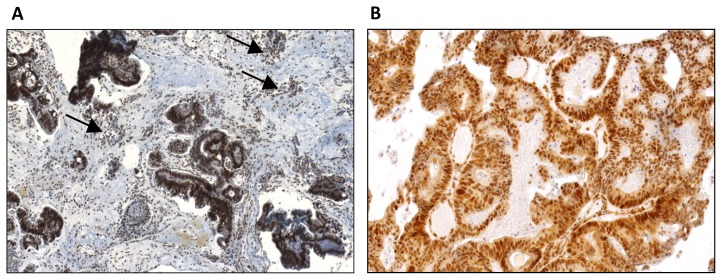
hMLH1 and hMSH2 expression in ITAC. (**A**) Nuclear immunoreactivity for hMLH1 in a papillary type of ITAC both in tumor cells and lymphocytes cells (arrows); (**B**) Nuclear immunoreactivity for hMSH2 in a papillary type of ITAC in tumor cells.

The MSI analysis was performed using antibodies against the following markers: hMLH1 (clone G168-728, Pharmingen International; 1:70 diluted); and hMSH2 (clone G219-1129, Pharmingen International; 1:100 diluted) and using the BenchMark ULTRA ICH/ISH Staininig Module, according to ULTRAView universal DAB detection kit and procedure. Loss of expression in tumors cells was considered when normal nuclear staining in adjacent non neoplastic cells or lymphocytes cells was observed (Figure 2).

### 3.3. KRAS and BRAF Analysis

Tumor-rich areas marked by the pathologist on hematoxylin and eosin histologic sections were manually cored and collected in a microtube for genetic testing. Tumor DNA was extracted from paraffin-embedded tissue samples using Qiagen extraction kits (QIAamp DNA FFPE tissue kit) according to the manufacturer’s recommendations. Special care was taken to obtain high-quality DNA from the fixed paraffin-embedded tissues. With this protocol, most fixed, paraffin embedded tissue samples yielded DNA of relatively good quality measured by Nanodrop.

*KRAS* exon 2 and *BRAF* exon 15 were PCR-amplified using a Rotor-Gene 6000™ instrument (Qiagen, Courtaboeuf, France) and a LightCycler 480 High Resolution Melting (HRM) Master Reaction Mix (Roche Diagnostics, France). Each 20 μL reaction volume consisted of 25 ng purified genomic DNA, 10 μL reaction mix, 3.0 mmol/L MgCl_2_ and 0.25 μmol/L of each forward and reverse primer. The primer sequences are as follows:

KRAS-F: 5′-GGCCTGCTGAAAATGACTGAA-3′;
KRAS-R: 5′-ATTAGCTGTATCGTCAAGGCACTC-3′;
BRAF-F: 5′-ATGAAGACCTCACAGTAAAAAJAGG-3′;
BRAF-R: 5′-AGCAGCATCTTAGGGCCAAA-3′.

The cycling conditions were identical for all amplifications and were as follows: 95 °C for 5 min, followed by 50 cycles of 95 °C for 15 s, 63 °C for 25 s with an initial 11 touchdown cycles (0.5 °C/cycle), and 72 °C for 25 s. The melting conditions included one 95 °C cycle for 1 min, one 40 °C cycle for 1 min and one 65 °C cycle for 2 s, followed by a gradual increase from 65 to 95 °C at 0.1 °C/s. All samples were tested in duplicate. The HRM data were analyzed using Rotor-Gene 6000 software (v.1.7; France). For each sample, the normalized melting curves were evaluated, and the samples were compared with the wild-type sample controls in a deduced difference plot. Significant deviations from the horizontal line relative to the spread of the wild-type controls were indicative of sequence changes within the analyzed amplicon. Samples with distinct melting curves compared with the wild-type allele were recorded as positive mutations. Mutation identifications were then determined using Cobas^®^ 4800 BRAF V600, KRAS and EGFR mutation tests (Roche Applied Sciences, France) according to the manufacturer’s instructions. All samples were analyzed in duplicate.

### 3.4. Statistical Analysis

Possible correlations between genetic and clinical parameters were statistically analysed by SPSS 12.0 software for Windows (SPSS Inc., Chicago, IL, USA), using the Pearson chi-square test and Fischer’s exact test. Kaplan–Meier analysis was performed for estimation of survival, comparing survival distributions through a log-rank test. *p* < 0.05 values were considered significant.

## 4. Conclusions

In conclusion, the results of this genetic and phenotypic analysis confirmed the many morphological and molecular similarities between ITAC and colorectal cancer. The vast majority of ITACs resembled colorectal cancer, showing deregulation of *KRAS/BRAF* genes that are involved in the early phases of colorectal cancer development. *KRAS* mutations were observed in 42.9% of ITAC, but no significant differences were observed in clinicopathological parameters based on the *KRAS* genotype. Surprisingly, however, *KRAS* mutations predicted good prognosis in ITAC. *BRAF* mutations were found in 1 out 28 ITACs tested. However owing to PCR inhibition, 15 cases were excluded from the statistical analyses, and our low number of cases could cause shortcomings, so that these results require confirmation in a larger cohort. Current ITAC treatment approaches include surgery, which may be accompanied by radiotherapy and chemotherapy. However, the 5-year cumulative survival rate is around 40% and therefore new therapeutic approaches are needed to improve this prognosis. Anti-EGFR treatments that have been developed for colon cancer are promising. Current guidelines state that patients with metastatic colorectal carcinomas being considered for EGFR therapies should be tested for *KRAS* mutations, independently of the EGFR expression level. Our results showed that in ITAC, *KRAS* mutations were frequent and EGFR overexpression was not rare. Hence, EGFR directed molecular treatments could be investigated in a subset of patients affected by ITAC, alone or in combination with chemotherapy, and *KRAS* mutation analysis could be used to preclude patients from receiving such treatment.

## Figures and Tables

**Table 1:** Clinical features of 43 patients with intestinal-type adenocarcinoma (ITAC).

Characteristics	Number of patients (%)
Sex	
Male	42 (98)
Female	1 (2)

Age (years)	
Range	39–80
Median	66

Stage	
T1	1 (2)
T2	16 (37)
T3	9 (21)
T4	17 (40)

N	
Yes	3 (7)
No	39 (91)
NA	1 (2)

M	
Yes	6 (14)
No	36 (84)
NA	1 (2)

Histological type	
Papillary	3 (7)
Colonic	25 (58.1)
Solid	5 (11.6)
Mucinous	10 (23.3)

Wood dust exposure	
Yes	26 (60)
No	9 (21)
NA	8 (19)

Recurrence	
Yes	24 (56)
No	17 (40)
NA	2 (4)

Outcome	
Alive	16 (37)
Died of disease	16 (37)
Died of other causes	9 (21)
NA	2 (5)

NA, not available.

**Table 2:** Demographic, clinicopathologic and, genetic alterations, and follow-up data of ITAC patients.

Case	T	N	M	Wood exposure	Histology	EGFR overexpression	MLH1/MSH2 expression	*KRAS* exon 2	*BRAF* exon 15
1	T4	No	No	No	Solid	No	Yes	Wild-type	Wild-type
2	T2	No	No	Yes	Colonic	No	Yes	Not amplified	Not amplified
3	T4	Yes	Yes	NA	Solid	No	Yes	Wild-type	Wild-type
4	T4	No	No	No	Colonic	Yes	Yes	Mutation	Wild-type
5	T4	No	No	Na	Mucinous	No	Yes	Not amplified	Not amplified
6	T2	No	No	Yes	Colonic	No	Yes	Mutation	Wild-type
7	T2	No	No	Yes	Colonic	No	Yes	Mutation	Wild-type
8	T4	No	No	Yes	Colonic	No	Yes	Not amplified	Not amplified
9	T4	No	Yes	No	Mucinous	No	Yes	Not amplified	Not amplified
10	T2	No	No	No	Colonic	Yes	Yes	Wild-type	Wild-type
11	T2	No	No	No	Mucinous	No	Yes	Wild-type	Wild-type
12	T2	No	No	Yes	Colonic	No	Yes	Wild-type	Wild-type
13	T2	No	No	NA	Colonic	Yes	Yes	Mutation	Wild-type
14	T2	No	No	Yes	Papillary	No	Yes	Mutation	Wild-type
15	T3	No	No	Yes	Colonic	No	Yes	Mutation	Wild-type
16	T3	No	No	Yes	Colonic	No	Yes	Wild-type	Wild-type
17	T3	No	No	No	Mucinous	No	Yes	Not amplified	Not amplified
18	T2	No	No	Yes	Colonic	No	Yes	Wild-type	Wild-type
19	T2	Yes	No	Yes	Solid	No	Yes	Mutation	Wild-type
20	T2	No	No	Yes	Colonic	No	Yes	Not amplified	Not amplified
21	T4	No	No	Yes	Mucinous	No	Yes	Not amplified	Not amplified
22	T2	No	No	Yes	Colonic	No	Yes	Wild-type	Mutation
23	T2	No	No	Yes	Mucinous	No	Yes	Not amplified	Wild-type
24	T3	No	No	Yes	Mucinous	No	Yes	Mutation	Wild-type
25	T3	No	No	Yes	Colonic	Yes	Yes	Not amplified	Not amplified
26	T2	No	No	Yes	Papillary	Yes	Yes	Not amplified	Not amplified
27	T2	Yes	Yes	Yes	Mucinous	No	Yes	Mutation	Wild-type
28	T4	No	No	No	Colonic	Yes	Yes	Wild-type	Wild-type
29	T4	No	No	No	Colonic	Yes	Yes	Wild-type	Wild-type
30	T3	No	No	Yes	Colonic	Yes	Yes	Wild-type	Wild-type
31	T4	No	No	NA	Solid	No	Yes	Wild-type	Wild-type
32	T1	No	No	NA	Colonic	No	Yes	Wild-type	Wild-type
33	T4	No	Yes	Yes	Colonic	Yes	Yes	Wild-type	Wild-type
34	T4	No	No	Yes	Mucinous	No	Yes	Mutation	Wild-type
35	T4	No	Yes	NA	Solid	No	No evaluable	Not amplified	Not amplified
36	T4	No	No	Yes	Colonic	Yes	Yes	Wild-type	Wild-type
37	T3	No	No	Yes	Colonic	Yes	Yes	Not amplified	Not amplified
38	T2	No	No	NA	Colonic	No	Yes	Mutation	Wild-type
39	T4	No	Yes	Yes	Colonic	Yes	Yes	Not amplified	Not amplified
40	T3	No	No	Yes	Colonic	No	Yes	Not amplified	Not amplified
41	T4	NA	NA	NA	Mucinous	No	Yes	Mutation	Wild-type
42	T3	No	No	Yes	Colonic	Yes	Yes	Not amplified	Not amplified
43	T4	No	No	No	Papillary	No	Yes	Wild-type	Wild-type
